# Evaluation of the comparative efficacy of green lipped mussel plus krill oil extracts (EAB-277), *Biota orientalis* extracts or NSAIDs for the treatment of dogs with osteoarthritis associated pain: a blinded, placebo-controlled study

**DOI:** 10.3389/fvets.2024.1464549

**Published:** 2024-10-10

**Authors:** Naruepon Kampa, Duangdaun Kaenkangploo, Supranee Jitpean, Thanikul Srithunyarat, Suvaluk Seesupa, Somphong Hoisang, Karn Yongvanit, Phanthit Kamlangchai, Pongsatorn Tuchpramuk, B. Duncan X. Lascelles

**Affiliations:** ^1^Division of Surgery, Faculty of Veterinary Medicine, Khon Kaen University, Khon Kaen, Thailand; ^2^Veterinary Teaching Hospital, Faculty of Veterinary Medicine, Khon Kaen University, Khon Kaen, Thailand; ^3^Faculty of Veterinary Sciences, Mahasarakham University, Muang Mahasarakham, Thailand; ^4^Translational Research in Pain Program, Comparative Pain Research and Education Centre, Department of Clinical Sciences, College of Veterinary Medicine, North Carolina State University, Raleigh, NC, United States; ^5^Center for Translational Pain Research, Department of Anesthesiology, Duke University, Durham, NC, United States; ^6^Thurston Arthritis Center, UNC, Chapel Hill, NC, United States

**Keywords:** OA, marine based fatty acid, omega 3, NSAID, gait analysis, PVF

## Abstract

**Introduction:**

With little to no regulation of the supplement markets and a paucity of quality information regarding clinical utility of individual marketed supplements, it is difficult for veterinarians to provide any evidence-based recommendations to owners. The current study aimed to provide clinically useful comparative efficacy data on certain marketed supplements.

**Methods:**

Using a prospective, block-randomized, double-blinded, placebo-controlled design, one hundred and one pet dogs with clinical hip OA-associated pain with one side worse than the other (index limb) were randomly assigned to one of four treatment groups: Green lipped Mussel plus Krill oil extracts (Antinol® Rapid, EAB-277); *Biota orientalis* extracts (4CYTE™ Epiitalis® Forte); an NSAID (meloxicam); or placebo (sunflower oil). Peak vertical force (PVF, expressed as a percentage of bodyweight) of the index limb, orthopedic assessment score (OAS) and hematology and blood chemistry values were evaluated before treatment (week 0), at 2, 4 and 6 weeks during treatment.

**Results:**

At 6 weeks, the changes from baseline in PVF of the index limb in the EAB-277 and meloxicam groups were significantly greater than the change in the placebo and 4CYTE™ groups, and the placebo and 4CYTE groups were not different from each other. At 6 weeks, there were significant differences between the groups for overall OAS scores with the lowest scores (least impairment) in the EAB-277 and meloxicam groups, followed by the 4CYTE group and then the placebo group.

**Discussion:**

Results of this study indicate that meloxicam and EAB-277 have significant objectively measured benefits in managing OA-related pain in dogs compared to placebo, but 4CYTE does not differ from placebo.

## Introduction

Osteoarthritis (OA) is characterized by the progressive deterioration of one or more of the component tissues of the joint. This deterioration can be associated with pain and this pain has widespread, cumulative negative effects on multiple domains including mobility, the ability to perform the activities of daily living, musculoskeletal health and sensory processing which together negatively impact a dog’s quality of life (1, 2). Recent data suggest that approximately 37% of dogs in the population may exhibit OA and related clinical signs due to pain (3). Additionally, new information has demonstrated that radiographically visible OA is very common in young dogs (8 months-4 years) with approximately 40% being affected radiographically and ~ 16% having associated pain of a moderate level or greater (4). Clearly, OA and associated pain is a common condition of dogs.

Managing OA pain in dogs typically involves a multimodal approach, including pain management, weight management, physical therapy, nutritional support and potentially surgical intervention in severe cases (5, 6). Non-steroidal anti-inflammatory drugs (NSAIDs) or anti-nerve growth factor monoclonal antibodies are recommend as the first line pharmacological therapy for dogs with chronic pain (7, 8). Omega-3 fatty acids are also recommended as a first line non-pharmacological option, with non-omega-3 based supplements related to ‘tier 3’ (7, 8). Despite this recommendation, with so many supplements available, little to no regulation of the supplement markets and a paucity of quality information regarding clinical utility of individual marketed supplements, it is difficult for veterinarians to provide any evidence-based recommendations. The current study aimed to provide clinically useful comparative efficacy data on certain marketed supplements.

EAB-277 (Antinol Rapid®) is the proprietary combination of phospholipids extracted from krill oil together with lipid fractions from the Green Lipped Mussel (PCSO-524™). Several studies have shown an apparent benefit of PCSO-524 for canine OA pain (9–13). Krill oil, extracted from krill, a small red-colored crustacean inhabiting the Antarctic, has been suggested to have advantages over fish oil due to its higher phospholipid-bound EPA and DHA content. A study in humans revealed that krill oil alleviated pain symptoms in adults with mild knee discomfort (14). Additionally, a recent blinded, placebo-controlled study using objective outcome measures concluded EAB-277 showed effectiveness for OA pain in dogs (13).

Extracts from the plant *Biota orientalis* are commonly used in Chinese herbal medicine (15). In traditional medicine, it has been used in the treatment of liver diseases, bullous bronchitis, psoriasis, enuresis, amenorrhea, cystitis, uterine carcinomas, diarrhea, and rheumatism (16). Preclinical studies conducted *in vitro* and unspecified *in vivo* studies have revealed the antioxidant (17) and anti-inflammatory (18, 19). In a pilot work, the effectiveness of hydrolyzed oil extract from *Biota orientalis* seeds (hBO/Epiitalis®, Interpath Pty Ltd) was investigated in humans with knee pain due to OA and results suggested efficacy (20). Epiitalis® is a proprietary oil extract from the plant *Biota orientalis*. A pilot study of 4CYTE™ Epiitalis® Forte reported significant improvements in both objective measures of limb use and subjective quality of life questionnaire scores in a population of dogs with pre-existing lameness due to joint OA (21), however no control group was included in this open label study. In a subsequent study, 4CYTE Canine (containing Epiitalis® plus three marine-derived ingredients) was reported to be non-inferior to carprofen over a 28-day study in dogs with OA pain (22).

We hypothesized that the commercially available supplement preparations Antinol® Rapid and 4CYTE™ Epiitalis® Forte would have beneficial effects in treating OA pain in dogs as compared to placebo and benchmarked against a positive control, the NSAID meloxicam.

## Materials and methods

### Study design

This study was a prospective, block-randomized, double-blinded, placebo-controlled clinical trial in client-owned (pet) dogs. Approval for the study protocol was obtained from the Institutional Animal Care and Use Committee of Khon Kaen University (IACUC-KKU-53/64). Throughout the study duration, the dogs remained in the care of their owners. Prior to commencement, each owner received a detailed explanation of the study, and consent was obtained through signed consent forms. The study took place at the Veterinary Teaching Hospital (VTH), Faculty of Veterinary Medicine, Khon Kaen University (KKU), Thailand, spanning from 2021 to 2023.

### Sample size estimation

The sample size was estimated based on the change in peak vertical force (PVF) observed in a prior study evaluating the efficacy of EAB-277 in dogs with hip osteoarthritis (13). Changes in PVF after 4 weeks of treatment were + 3.90, +4.17, +3.08, and + 0.08 for the PCSO-524, EAB-277, Carprofen, and placebo groups, respectively. A type I error probability was set at 0.05 and power at 0.80 (1 minus the probability of type II error) were specified. Utilizing G*Power software (version 3.1.9.3, Heinrich Heine University Düsseldorf, Germany) for repeated measurement trials, it was determined that a sample size of 25 dogs per group was necessary.

### Animals

Pet dogs, regardless of breed or sex, were eligible for participation in the study if they met the following criteria: at least 18 months old, weighing at least 15 kg, having a body condition score ranging from 3 to 9 (on a 9-point scale system), and exhibiting hematology and blood chemistry values within normal ranges. Additionally, the medical history had to include disability as reported by their owners and considered by the veterinarian as being due to OA pain; dogs were required to have clinical signs of hindlimb lameness due to hip OA pain; hip joint pain during examination by a study veterinarian; and radiographic evidence of OA in one or both hip joints that were found to be painful upon examination. Furthermore, dogs were required to be capable of trotting across a force plate for gait analysis. The hindlimb with the lowest value of peak vertical force (PVF) was denoted as the index limb at the initial evaluation (see section on gait analysis).

Dogs were not eligible if: they could not trot across the force platform; were lame or impaired due to an orthopedic condition that was not OA, had undergone any joint surgery within the preceding 6 months, displayed clinically detectable neurological deficits or systemic diseases, or if they were pregnant or lactating bitches.

### Study protocol

The study protocol was written prior to the start of the study and agreed upon by all investigators. It was not publicly registered. Dogs were recruited to the VTH by outreach to local practitioners. Each dog underwent a full physical, orthopedic and neurological examination (conducted by SH), and the orthopedic assessment scores (OAS) were documented (Table 1). Radiographs of the hips were obtained and interpreted by a single radiologist (NK). Radiographic severity was evaluated based on established criteria outlined in previous publications (23) (Table 2).

**Table 1:** Assessment system used in the orthopedic evaluation (Orthopedic Assessment Scores, OAS) (24).

Criterion	Clinical evaluation
Lameness	1. Walks normally
	2. Slightly lame when walking
	3. Moderately lame when walking
	4. Severely lame when walking
	5. Reluctant to rise and will not walk more than five paces
Joint mobility	1. Full range of motion
	2. Mild limitation (10–20%) in range of motion; no crepitus
	3. Mild limitation (10–20%) in range of motion; with crepitus
	4. Moderate limitation (20–50%) in range of motion; with crepitus
	5. Severe limitation (>50%) in range of motion; with crepitus
Pain on palpation	1. None
	2. Mild signs; dog turns head in recognition
	3. Moderate signs; dog pulls limb away
	4. Severe signs; dog vocalizes or becomes aggressive
	5. Dog will not allow palpation
Weight-bearing	1. Equal on all limbs standing and walking
	2. Normal standing; favors affected limb when walking
	3. Partial weight-bearing standing and walking
	4. Partial weight-bearing standing; non-weight-bearing walking
	5. Non-weight-bearing standing and walking
Overall score of clinical condition	1. Not affected
	2. Mildly affected
	3. Moderately affected
	4. Severely affected
	5. Very severely affected

Each part of the OAS was scored and analyzed separately.

**Table 2:** Scoring system for the radiographic evidence of osteoarthritis (2)

Articulation	Radiographic sign	Score
Hip	Osteophytes and sclerosis absent	0 (none)
	Acetabular remodeling, Morgan line, slight neck remodeling and slight femoral head sclerosis	1 (mild)
	Acetabular remodeling and osteophytosis, neck remodeling, enthesiophytosis, and femoral head sclerosis	2 (moderate)
	Advanced acetabular and neck remodeling, severe osteophytosis and advanced femoral head sclerosis	3 (severe)

Prior to starting the study, dogs were required to undergo a 2-week washout period for NSAIDs and joint supplements, and a 4-week washout period for corticosteroids. Throughout the study, no additional analgesic therapies were allowed. The diet type and quantity, as well as the daily activities of the study dogs, were kept consistent throughout the study period.

Each dog and its owner made four visits to the hospital: one for screening and enrollment prior to treatment, and then at 2, 4 and 6 weeks post-treatment. During each visit, ground reaction force measurements of the hindlimbs were recorded, and orthopedic evaluations were performed. Samples for complete blood count and serum chemistry, as well as urine for urinalysis, were obtained at each time point. Dogs were permitted to withdraw from the study for any reason, at any time, at the discretion of the researchers, the attending veterinarian, or the owners. If dogs withdrew from the study, they received treatment as determined appropriate by the referring veterinarian.

### Treatment groups, allocation and blinding methods

Enrolled dogs were categorized into two groups (mild and moderate severity groupings) based on the severity of signs associated with OA pain using the overall orthopedic assessment score (see Table 1). Within each severity classification, dogs were randomly assigned to treatment groups. The allocation of treatments was carried out by the trial coordinator, who was not involved in assessing the dogs. Both the investigators collecting data and the dog owners were kept unaware of the treatment assignments. The trial coordinator provided guidance to the owners on the administration of treatments, including instructions on how and when to administer them. The treatments were provided in their original manufactured capsule or tablet form, distributed in unlabeled containers. The placebo consisted of capsules containing sunflower oil, prepared to match the appearance of EAB-277.

Using computer generated random numbers, dogs were randomly assigned to one of the four groups:

1. Antinol® Rapid (EAB-277) (Pharmalink International Co. Ltd.), administered orally at a dosage of 1 capsule per 10 kg body weight twice daily for a duration of 6 weeks.
2. 4CYTE™ Epiitalis® Forte gel (Interpath Co. Ltd.) administered once daily at the dose recommended by the manufacturer (1.0 mL for 10–20 kg, 1.5 mL for 20–30 kg, 2.0 mL for 30–40 kg and 2.5 mL for 40–50 kg) for a period of 6 weeks.
3. Meloxicam (Metacam®, Boeringher Ingelheim Co. Ltd.) administered orally at a dose of 0.2 mg/kg on the first day, followed by 0.1 mg/kg orally, every 24 h, for 6 weeks
4. Placebo capsules containing sunflower oil were administered at the same dosage as prescribed in group 1.

## Outcome measures

### Ground reaction force measurement: peak vertical force

Gait analysis was performed using dual in series biomechanical strain gage force plates (Advanced Mechanical Technology®, AMTI Model OR6-6, Watertown, MA, United States); 40 × 60 cm size each embedded in the middle of a 8-m-long walkway. Dogs were trotted across the force plates by trained handlers. The signals from the force plates were acquired and processed through dedicated gait analysis software (ToMoCoFPm, Toso System Inc.®, Saitama, Japan) and peak vertical force (PVF) values extracted. Velocity was measured by four laser sensors mounted 50 cm apart, spanning a distance on either side of the force plates. Velocity was limited to a range of 1.7–2.2 m/s and acceleration range within 0.5 m/s^2^ throughout the study. A video camera (Panasonic HC-V180, Panasonic, Japan) recorded each pass to confirm appropriate foot strikes of each limb. The valid trial was defined as the forelimb followed by the ipsilateral hindlimb striking the center of the force plate. The initial PVF value was reported in Newton meter (Nm), then was normalized to body weight, and expressed as a percentage of total body weight for each limb. The mean value of PVF at each evaluation time point was derived from the average of the first five valid trials collected. The hindlimb with the lowest value of PVF was denoted as the index limb at the initial evaluation (before treatment) and the index limb was followed for improvement of limb function during the study period.

### Orthopedic assessment scores

Following gait analysis at each time point, an orthopedic evaluation was conducted, and Orthopedic Assessment Scores (OAS) were documented. The OAS system, initially proposed by Moreau et al. (2) and later modified by McCarthy et al. (24), includes assessments of lameness, joint mobility, pain upon palpation, weight-bearing, and overall impact, with scoring criteria detailed in Table 1. Although it has not been formally defined or tested, a category change of ‘1’ is considered clinically relevant.

### Hematology and blood chemistry evaluations

A blood sample was collected from each dog before treatment and during every visit. Complete blood count (CBC) and serum biochemistry profiles were assessed. The serum biochemistry analysis consisted of blood urea nitrogen (BUN), creatinine, alanine aminotransferase (ALT), alkaline phosphatase (ALK), total protein, albumin, globulin, and the albumin:globulin ratio.

### Statistical analysis

Prior to treatment (week 0), the homogeneity of variables among groups was assessed. Categorical data such as sex, body condition score, affected limb side, affected joint, radiographic score, and OAS were analyzed using the Chi-square test. Continuous and ordinal data including age, body weight, lameness score, pain score, joint mobility score, weight-bearing score, overall score, and PVF index limb were evaluated using one-way analysis of variance (ANOVA) for normally distributed data and the Kruskal-Wallis test for non-normally distributed data or ordinal data. The experimental unit was each individual dog.

The primary outcome, PVF of the index limb (PVF_index_) expressed as a percentage of total bodyweight, was utilized to calculate changes in PVF_index_ at each time point relative to baseline (week 0) (deltaPVF_index_). The effect of treatment on PVF_index_, deltaPVF_index_, and OAS (including lameness score, pain score, joint mobility score, weight-bearing score, and overall score) was explored using linear mixed models with repeated measurements. Treatment group, visit time, and their interaction were considered as fixed factors, while the subject’s response measured at multiple time points was treated as a random factor with unstructured variance components. Simple effects between treatment groups at different time points and contrasts between visits within each group were examined using the CONTRAST options with Bonferroni adjustment. The minimal detectable change at the 95% confidence interval (MDC95), previously proposed by Moreau with a cutoff value of an increase in PVF >2.0% body weight was used to distinguish responders (25). All statistical analyses were performed using the STATA software (STATA v18, University licensed, StataCorp LLC, Texas, United States), and statistical significance was determined at a *p*-value of less than 0.05.

## Results

Following screening, a total of 101 dogs were included in the study, distributed across treatment groups as follows: 26 in the EAB-277 group, 25 in the 4CYTE™ group, 24 in the meloxicam group, and 26 in the placebo group. All enrolled dogs were included in all analyses. Of these, 66 were male and 35 were female, with average (mean ± SD) age, body weight, and body condition score (BCS) (median, range) of 5.23 ± 2.63 years, 32.83 ± 9.55 kg, and 4 (6), respectively. Ten breeds of dogs participated, including Alaskan Malamute, American Bully, Beagle, German Shepherd, Golden Retriever, Labrador Retriever, Samoyed, Siberian Husky, Thai Native, and mixed breeds. Golden and Labrador Retrievers were the predominant breeds, accounting for 41 and 21% of the total, respectively.

On clinical examination, 42 dogs predominantly exhibited lameness in the right hindlimb, while 59 dogs exhibited lameness in the left hindlimb. Of these, 54 dogs were classified as having mild OA, while 47 were classified as having moderate OA. Radiographic assessment revealed bilateral hip osteoarthritis (OA) lesions (radiographic score of hip ≥1) in 71 dogs and unilateral lesions in 30 dogs. Characteristics of the dogs, including sex, body condition score, affected limb side, unilateral or bilateral affection, radiographic severity score, OA classification, Orthopedic Assessment Scores (OAS), and PVF_index_ at baseline (week 0), are presented in Table 3. There were no significant differences between the four treatment groups (*p* > 0.05) for any variable. The hematology and blood chemistry values of all dogs were within normal limits during the study period of 6 weeks. There were no unexpected adverse events.

**Table 3:** Subject characteristics and data at prior treatment (week 0) of treatment groups and comparison.

Variable	EAB-277	4CYTE™	Meloxicam	Placebo	*p*- value
	*n* = 26	*n* = 25	*n* = 24	*n* = 26	
Categorical variables*					
Sex					
Male	17	17	17	15	0.784
Female	9	8	7	11	
BCS					
3	1	1	1	0	0.955
4	16	16	16	16	
5	6	5	5	5	
7	1	2	2	4	
9	2	1	0	1	
Side of affected limb					
Right	14	7	9	12	0.273
Left	12	18	15	14	
Affected joint					
Unilateral	8	7	7	8	0.996
Bilateral	18	18	17	18	
Radiographic score (index limb)					
1	10	8	6	8	0.954
2	9	9	8	9	
3	7	8	10	9	
Radiographic score (contralateral limb)					
0	8	7	7	8	0.976
1	7	5	4	4	
2	7	8	6	7	
3	4	5	7	7	
OA classification					
Mild OA	16	12	12	14	0.777
Moderate OA	10	13	12	12	
Continuous variables** (mean ± SD)					
Age (years)	4.94 ± 2.52	5.24 ± 2.68	5.58 ± 2.81	5.19 ± 2.64	0.843
Body weight (kg)	32.06 ± 8.96	33.22 ± 8.55	33.13 ± 8.70	34.21 ± 9.27	0.804
PVF index limb	58.44 ± 7.13	61.34 ± 7.99	59.22 ± 7.45	60.46 ± 8.82	0.568
Non-parametric variable*** (median, range)					
Lameness score	3.0 (2.0)	2.0 (3.0)	3.0 (3.0)	2.0 (2.0)	0.297
Pain score	2.0 (3.0)	2.0 (2.0)	2.0 (2.0)	2.0 (3.0)	0.379
Joint mobility score	2.0 (3.0)	2.0 (2.0)	2.0 (2.0)	2.0 (2.0)	0.878
Bearing score	2.0 (2.0)	2.0 (2.0)	2.0 (2.0)	2.0 (1.0)	0.306
Overall score	2.0 (2.0)	2.0 (1.0)	2.5 (2.0)	2.0 (1.0)	0.886

BCS, body condition score; OAS, Orthopedic Assessment Scores. *Chi-square tests. **One-way ANOVA. ***Kruskal-Wallis tests.

### Force plate gait analysis: peak vertical force

Velocity at each time point showed no differences either between or within groups (Supplementary Table S1).

**Figure 1:**
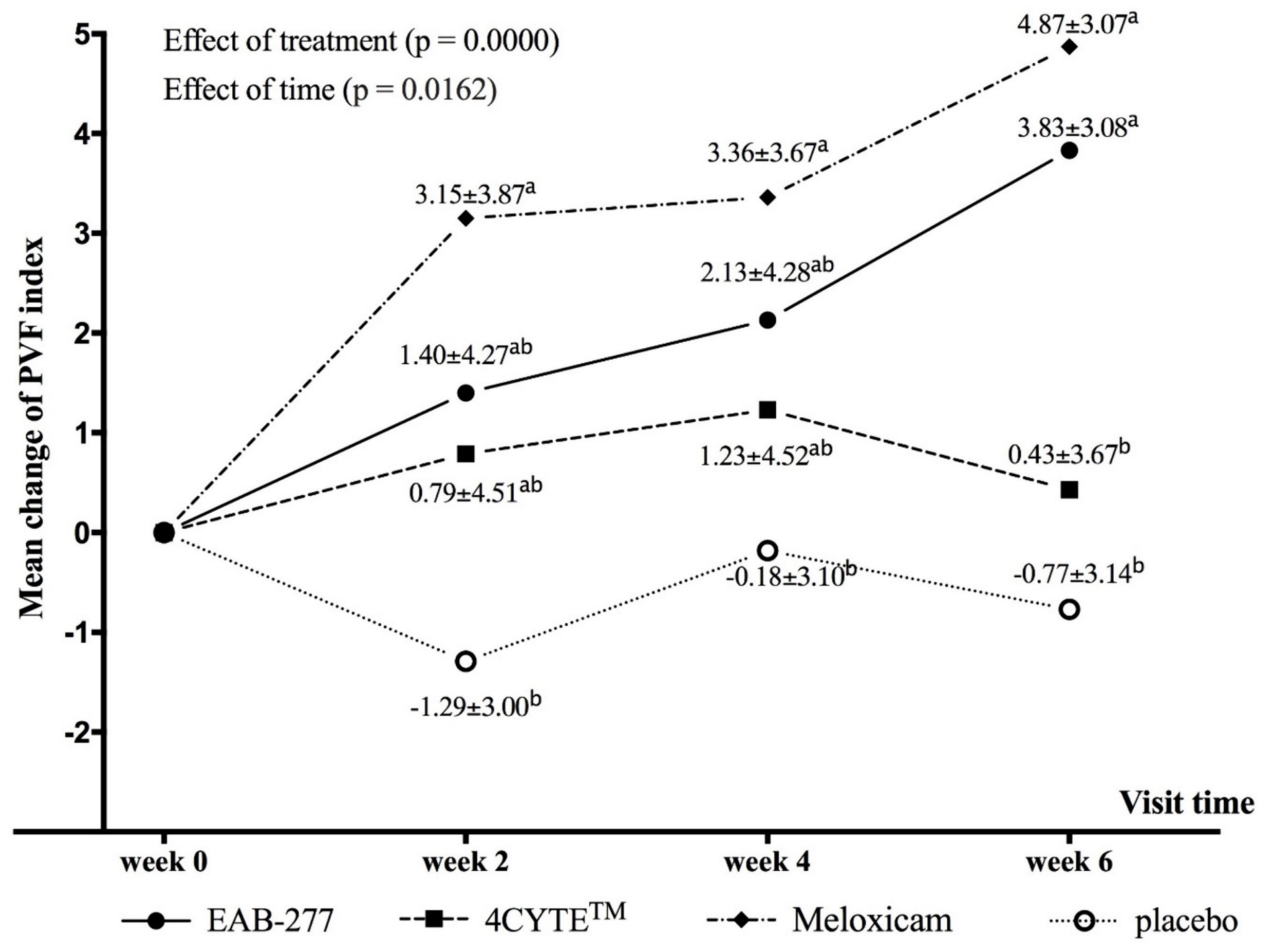
Graphical representation of the mean (±standard deviation) change from baseline for PVF (deltaPVF_index_) in each group during the study period. Different in lower letter superscript (a, b) indicate significant differences between groups for mean deltaPVF_index_.

There was a notable and significant overall effect of treatment (*p* < 0.001) and time (*p* = 0.016) on the change in the primary outcome measure PVF_index_. Specifically, the dogs in the EAB-277 and meloxicam groups showed increases in the mean deltaPVF_index_ from week 0 over time, whereas the 4CYTE™ group demonstrated minimal change and the placebo group exhibited no change throughout the study period. By week 2 post-treatment, dogs in the meloxicam group showed a significant increase in PVF_index_ compared to pre-treatment levels (Supplementary Table S2), with a mean deltaPVF_index_ (3.15 ± 3.87) that was significantly higher than in the placebo group (−1.29 ± 3.00) (Table 4; Figure 1). Following 4 weeks of treatment, both the EAB-277 and meloxicam groups showed a significant increase in PVF_index_ compared to baseline (Supplementary Table S2). The mean deltaPVF_index_ was 2.13 ± 4.28 in the EAB-277 group, 1.23 ± 4.52 in the 4CYTE™ group, and 3.36 ± 3.67 in the meloxicam group, with the latter significantly higher than the placebo group (−0.18 ± 3.10) (Table 4; Figure 1). At the final observation point (week 6), both the EAB-277 and meloxicam groups had significantly greater changes in PVF_index_ compared to baseline, similar to the results at week 4 (Supplementary Table S2). The mean deltaPVF_index_ for the EAB-277 (3.83 ± 3.08) and meloxicam (4.87 ± 3.07) groups was significantly higher than that of the 4CYTE™ group (0.43 ± 3.67) and the placebo group (−0.77 ± 3.14) (Table 4; Figure 1). Using the MDC95 as a cut-off value ±2.0% PVF of body weight, the percentage of responders in each treatment group (EAB-277, 4CYTETM, Meloxicam and placebo) was 69.23, 40.00, 79.19, and 7.69%, respectively (Table 5).

**Table 4:** The mean deltaPVF_index_ ± Standard Deviation [95% confident interval] for dogs in each group at 2, 4 and 6 weeks following initiation of treatment.

Visit time	EAB-277	4CYTE™	Meloxicam	Placebo
	*n* = 26	*n* = 25	*n* = 24	*n* = 26
Week 0 (PVF)	58.44 ± 7.13	61.34 ± 7.99	59.22 ± 7.45	60.46 ± 8.82
Week 2 mean change	1.40 ± 4.27 ^a,b^	0.79 ± 4.51 ^a,b^	3.15 ± 3.87* ^a^	−1.29 ± 3.00 ^b^
	[−0.04, 2.84]	[−0.68, 2.25]	[1.65, 4.64]	[−2.73, 0.14]
Week 4 mean change	2.13 ± 4.28* ^a,b^	1.23 ± 4.52 ^a,b^	3.36 ± 3.67* ^a^	−0.18 ± 3.10 ^b^
	[0.57, 3.45]	[−0.24, 2.69]	[1.86, 4.86]	[−1.61, 1.26]
Week 6 mean change	3.83 ± 3.08* ^a^	0.43 ± 3.67 ^b^	4.87 ± 3.07* ^a^	−0.77 ± 3.1 ^b^
	[2.28, 5.17]	[−1.03, 1.91]	[3.36, 6.37]	[−2.22, 0.67]

Week 0 (prior treatment) absolute PVF values are also shown. *The 95% confident interval of estimated mean difference (deltaPVF_index_) not covered the zero value indicates that the value of the mean change in PVF of the index limb was significantly different (*p* < 0.05) from the week 0 within that treatment group. Between group comparisons are shown in Figure 1. Differences in lower letter superscript (a, b) indicate significant differences between groups for mean deltaPVF_index_.

**Table 5:** Percentage of responders (PVF change >2%) and non-responders (PVF change <2%) in each treatment group (EAB-277, 4CYTETM, Meloxicam and placebo) at week 6 after treatment, with a comparison between groups.

Group	Responders	Nonresponders	*p*- value*
EAB-277	69.23 (18/26) [48.21–85.67]	30.77 (8/26) [14.32–51.78]	<0.001
4CYTE™	40.00 (10/25) [21.12–61.33]	60.00 (15/25) [38.66–78.87]	
Meloxicam	79.17 (19/24) [57.84–92.86]	20.83 (5/24) [7.13–42.15]	
placebo	7.69 (2/26) [0.94–25.13]	92.31 (24/26) [74.86–99.05]	

Data are presented in % (number of dogs responding/total number of dogs) (95% confidence intervals). *Analysis by Chi square test.

### Orthopedic assessment scores

The lameness scores in the EAB-277 and Meloxicam groups exhibited significant decreases compared to pre-treatment levels, while scores in the 4CYTE™and placebo groups remained unchanged throughout the study period (Supplementary Table S3). Additionally, the pain scores in the Meloxicam group were consistently lower at all visits compared to pre-treatment, with the lowest scores observed during weeks 4 and 6 following treatment (Supplementary Table S4). Joint mobility scores significantly decreased in the EAB-277, 4CYTE™, and Meloxicam groups at weeks 2, 4, and 6 post-treatment (Supplementary Table S5). Similarly, bearing scores significantly decreased at weeks 2, 4, and 6 post-treatment compared to pre-treatment levels in the EAB-277 and Meloxicam groups (Supplementary Table S6).

Finally, the overall scores of the EAB-277 and Meloxicam groups showed significant decreases compared to pre-treatment levels, with both groups achieving their lowest scores at 6 weeks post-treatment (Table 6). At 6 weeks, there were significant differences between the groups, with scores being lowest (decreased clinical signs) in the EAB-277, 4CYTE and meloxicam groups compared to the placebo group. However, the degree of change in overall scores was quite small, likely reflecting the subjective nature of the assessments and the very coarse scale.

**Table 6:** The ‘overall scores’ of the orthopedic assessment score (median, range) for the treatment groups prior to treatment (week 0), week 2, 4 and 6 after treatment.

Visit time	EAB-277	4CYTE™	Meloxicam	placebo	*p*- value for between group comparisons
	*n* = 26	*n* = 25	*n* = 24	*n* = 26	
week 0	3.0 (2.0)	2.0 (3.0)	3.0 (3.0)	2.0 (2.0)	0.797
week 2	2.0 (3.0)	2.0 (1.0)	2.0 (1.0)*	2.0 (1.0)	0.997
week 4	2.0 (2.0)*	2.0 (1.0)	2.0 (2.0)*	2.0 (1.0)	0.453
week 6	2.0 (2.0)^a^*	2.0 (2.0)^a^	2.0 (2.0)^a^*	2.0 (2.0)^b^	0.027

^a,b^Different in lower letter superscript indicates significant differences between groups at time point. *Indicates the score in point time (week) significantly different (*p* < 0.05) from the value of week 0 in each treatment group.

## Discussion

This study selected dogs with hip joint osteoarthritis, with one leg more affected than the other, and used objective gait analysis – measurement of the ground reaction force peak vertical force – to assess the efficacy of two supplements compared to the NSAID meloxicam and to placebo, over a 6-week period. Overall, we found PVF increased over time (limb use improved) in both the EAB-277 and Meloxicam groups, whereas there was minimal improvement in the 4CYTE™ group and no change in the placebo group throughout the study. Positive effects were seen earliest in the meloxicam group (by week 2) and then in the EAB-277 group (by week 4). Conversely, 4CYTE™ and placebo did not exhibit positive treatment effects based on PVF measurements. Interestingly, at both week 4 and 6 post-treatment, the change in PVF for EAB-277 was similar to that of the Meloxicam group. Overall, the results show a clear benefit of EAB-277 and meloxicam in improving limb use in dogs over a 6-week period. In this study, both a positive control (the NSAID, meloxicam) and a negative control (placebo) were included to contextualize PVF changes in the other groups.

The results from gait analysis were supported by the subjective assessments across lameness, pain, joint mobility and weight-bearing scores. As well as the improvements in these parameters seen in the EAB-277 and meloxicam groups over time, joint mobility and weight bearing were assessed as being significantly improved compared to baseline in the 4CYTE™ at 6 weeks. However, across these parameters, only the pain score and the overall assessment scores showed significant group effects, favoring EAB-277 and meloxicam at week 6 for pain, and favoring all three treatment groups versus placebo for the overall score. Overall, the findings suggest potential benefits of EAB-277, 4CYTE™, and meloxicam in managing OA-related pain in dogs, as evaluated by the OAS.

In this study, we found no improvement of the objective assessment of GRFs with 4CYTE™. One previous study demonstrated significant improvements in both objective measures of limb use (TPI% [total pressure index]) using a pressure sensitive mat (GAITRite® Portable Walkway System) and subjective quality of life questionnaire scores (HCPI) in dogs with pre-existing lameness due to joint OA (21). However, this open-label study did not include a control group which makes it impossible to assess whether the changes seen were truly due to treatment, or the natural variation in impact of pain over time. In contrast, our study was a randomized, placebo-controlled trial that included both positive and negative control groups. Although the different gait analysis system was used for the objective assessment, both placebo and 4CYTE™ group’s PVF showed no significant change after the study was completed (6 weeks).

Our results regarding the efficacy of meloxicam, an NSAID, align with previous studies (1, 26–28). Meloxicam exhibited a rapid response in terms of increasing limb use (as measured by ground reaction forces), with significant improvement observed within 2 weeks of treatment evidenced by a PVF change of 3.15 ± 3.87. Our currently reported results for meloxicam are also similar to a study in dogs treated with carprofen for 2 weeks where a change in PVF (%BW) of 3.2 ± 0.8 (significant improvement) was seen (29), and similar to those from another OA study (9) involving Firocoxib, where the PVF change in the index limb was 3.03 ± 4.67 and 3.25 ± 4.13 after 2 and 4 weeks of treatment, respectively (9). The results of EAB-277 in this study were similar to those of the previous studies (13); the PVF change after 6 weeks of treatment was 3.83 ± 3.08, slightly lower than the 4.45 ± 4.23 observed in the previous study.

No work has been done to define the minimal clinically important difference (MCID) with respect to ground reaction forces. We are working on defining the MCID for change in PVF (in separate work) in dogs with multi-joint OA pain. In this study, responder analysis was evaluated using a previously determined cut-off value of ±2.0% PVF change (25, 30). Meloxicam had the highest percentage of response rate at 79.19%, followed by EAB-277 at 69.23%, 4CYTE™ at 40.00%, and placebo at 7.69%.

Overall, our results clearly indicated little to no positive effects associated with placebo. Further, the results from the positive and negative control groups give us confidence in interpreting the effects of administration of each of the supplements we evaluated, EAB-277 and 4CYTE™: the changes observed with meloxicam and EAB-277 were significantly different from the placebo group, strongly suggesting a clinically significant improvement. Given our inclusion criteria and the results in our positive and negative control groups, we believe our results are generalizable to the broader population of dogs with OA pain.

Our study had several limitations. Although clearly recommended in current pain management guidelines (7, 31), our study did not employ clinical metrology instruments (CMIs), or client reported outcome measures (CROMs) for assessing OA pain. There are several CMIs that have been developed, validated, and reported for measuring the severity of OA in dogs such as the Liverpool Osteoarthritis in Dogs (LOAD) instrument (32), the Canine Brief Pain Inventory (CBPI) (33), the Helsinki Chronic Pain Index (HCPI) (34). Owners must complete questionnaires, necessitating their understanding of the questions, which should also align with the local culture and context. A recent study in Thailand (9) that employed the CBPI suggested that the translated version might not have been fully comprehended. Our pilot experience with the LOAD indicated that, even after translation, the questions might not have been suitable for the Thai culture. Ideally, each CMI should be validated +/− adapted for each new language and culture. Therefore, CMIs were not used in this study as none have been validated in the Thai language and culture. Unlike CMIs, ground reaction forces (GRFs) measured using a force plate provide an objective assessment and have been utilized as a proxy measure of joint pain in dogs with appendicular joint OA (29, 35–39). Additionally, the duration of the study was only 6 weeks and it is possible that over longer durations of administration of supplements, greater effects may be seen. Extending the study duration may provide more comprehensive information about supplements’ effects on OA pain, however our results clearly indicate positive effects for EAB-277, but not 4CYTE, over a 6 week period. Finally, many times supplements are used together with NSAIDs, but we did not have a group evaluating combined treatment. Future research should evaluate the combination of EAB-277 with an NSAID to test for potential synergistic effects in multimodal therapy management.

## Conclusion

In dogs with painful OA, we found that PVF increased over time (indicating improved limb use) in both the EAB-277 and meloxicam groups, while there was minimal improvement in either the placebo or 4CYTE™ group. At 6 weeks there were significant differences between the groups in improvement in limb use, with improvement in the meloxicam and EAB-277 groups being significantly greater than in the placebo and 4CYTE groups. These results, combined with the subjective orthopedic assessments of lameness, pain, joint mobility, and weight-bearing scores, suggest that meloxicam and EAB-277 have clear benefits in managing OA-related pain in dogs, with equivocal evidence for a beneficial effect of 4CYTE™.

## Data Availability

The raw data supporting the conclusions of this article will be made available by the authors, without undue reservation.
