# Supplementary material for: Evaluation of the comparative efficacy of green lipped mussel plus krill oil extracts (EAB-277), Biota orientalis extracts or NSAIDs for the treatment of dogs with osteoarthritis associated pain: a blinded, placebo-controlled study
Source: Front Vet Sci. 2024 Oct 10;11:1464549. doi: 10.3389/fvets.2024.1464549 (PMC11500327; doi:10.3389/fvets.2024.1464549)
Supplement: Supplementary file 1 [file Data_Sheet_1.pdf]

Supplementary material

Supplementary Table 1 The velocity of 4 groups of treatment at prior treatment (week 0), week 2, 4 and 6 after treatment

Visit time	EAB-277 n=26	4CYTE™ n=25	Meloxicam n=24	placebo n=26	p-value (between group treatment effect)
week 0	2.07±0.13	2.08±0.15	2.07±0.16	2.06±0.12	0.950
week 2	2.08±0.10	2.11±0.13	2.06±0.13	2.04±0.15	0.378
week 4	2.05±0.15	2.05±0.16	2.08±0.11	2.04±0.15	0.763
week 6	2.11±0.11	2.07±0.14	2.09±0.11	2.05±0.15	0.476
p-value (time effect)	0.147	0.216	0.714	0.897	

Supplementary Table 2 Average of the absolute (mean±standard deviation) PVF values of 4 groups of treatment at prior treatment (week 0), weeks 2, 4 and 6 after treatment

Visit time	EAB-277 n=26	4CYTE™ n=25	Meloxicam n=24	placebo n=26	p-value (between group treatment effect)
week 0	58.44±7.13	61.34±7.99	59.22±7.45	60.46±8.82	0.592
week 2	59.84±8.20	62.13±7.19	62.37±7.69*	59.16±9.82	0.386
week 4	60.46±8.67*	62.56±8.50	62.58±7.60*	60.28±8.23	0.583
week 6	62.17±7.76*	61.77±7.60	64.09±6.37*	59.69±7.99	0.263

* indicates PVF of the index limb is significantly different ($p < 0.05$) from the value of week 0 within a given treatment group.

Supplementary Table 3 The lameness score of 4 group of treatment at prior treatment (week 0), week 2, 4 and 6 after treatment

Visit time	EAB-277 n=26	4CYTE™ n=25	Meloxicam n=24	placebo n=26	p-value (between group treatment effect)
week 0	2.65±0.56	2.36±0.70	2.63±0.65	2.46±0.58	0.259
week 2	2.50±0.58	2.36±0.49	2.25±0.53*	2.35±0.63	0.512
week 4	2.31±0.62*	2.32±0.69	2.08±0.41*	2.27±0.67	0.475
week 6	2.19±0.49*	2.24±0.66	2.04±0.55*	2.38±0.70	0.268

* indicates the score is significantly different ($p < 0.05$) from the value of week 0 within a given treatment group.

Supplementary Table 4 The pain score of 4 group of treatment at prior treatment (week 0), week 2, 4 and 6 after treatment

Visit time	EAB-277 n=26	4CYTE™ n=25	Meloxicam n=24	placebo n=26	p-value (between group treatment effect)
week 0	2.12±0.91	1.96±0.79	2.38±0.65	2.15±0.88	0.369
week 2	1.96±0.87	1.80±0.82	1.71±0.69*	2.12±0.82	0.251
week 4	$1.92±0.84^{ab}$	$1.76±0.72^{ab}$	$1.46±0.59^{a*}$	$2.08±0.84^{b}$	0.021
week 6	$1.69±0.55^{ab*}$	$1.88±0.67^{a}$	$1.33±0.48^{b*}$	$1.96±0.82^{a}$	0.009

^{a,b} Different in lower letter superscript indicate significant differences between groups at time point.

* indicates the score is significantly different ($p < 0.05$) from the value of week 0 within a given treatment group.

Supplementary Table 5 The joint mobility score of 4 group of treatment at prior treatment (week 0), week 2, 4 and 6 after treatment

Visit time	EAB-277 n=26	4CYTE™ n=25	Meloxicam n=24	placebo n=26	p-value (between group treatment effect)
week 0	2.35±0.69	2.48±0.71	2.46±0.66	2.35±0.56	0.811
week 2	2.08±0.48*	2.16±0.55*	2.04±0.62*	2.27±0.60	0.529
week 4	2.08±0.48*	2.16±0.55*	1.96±0.55*	2.23±0.51	0.348
week 6	2.00±0.69*	2.12±0.60*	1.88±0.54*	2.15±0.37	0.235

* indicates the score is significantly different ($p < 0.05$) from the value of week 0 within a given treatment group.

Supplementary Table 6 The bearing score of 4 group of treatment at prior treatment (week 0), week 2, 4 and 6 after treatment

Visit time	EAB-277 n=26	4CYTE™ n=25	Meloxicam n=24	placebo n=26	p-value (between group treatment effect)
week 0	1.77±0.51	1.60±0.58	1.88±0.54	1.58±0.50	0.181
week 2	1.58±0.58*	1.48±0.51	1.42±0.50*	1.58±0.50	0.616
week 4	1.58±0.58*	1.44±0.51	1.38±0.49*	1.46±0.51	0.532
week 6	1.42±0.50*	1.44±0.51*	1.25±0.44*	1.54±0.51	0.263

* indicates the score is significantly different ($p < 0.05$) from the value of week 0 within a given treatment group.
